# Beyond Tachycardia-Induced Cardiomyopathy: *ABCC9*-Related Dilated Cardiomyopathy and Familial Atrial Fibrillation

**DOI:** 10.1016/j.jaccas.2025.105302

**Published:** 2025-09-09

**Authors:** Alexander Kong, Teresa Bernardes, Elona Rrapo-Kaso, Alexies Ramirez, Steve Carlan

**Affiliations:** a Orlando Health Heart and Vascular Institute, Orlando Health, Orlando, Florida, USA; b Section of Cardiology, Orlando VA Healthcare System, Orlando, Florida, USA; c Department of Internal Medicine, University of Central Florida College of Medicine, Orlando, Florida, USA; d Department of Internal Medicine, Orlando Regional Medical Center, Orlando, Florida, USA; e Division of Academic Affairs and Research, Orlando Regional Medical Center, Orlando, Florida, USA

**Keywords:** *ABCC9*, atrial fibrillation, dilated cardiomyopathy, genetic testing, prolonged QT, tachycardia-induced cardiomyopathy

## Abstract

**Background:**

Tachycardia-induced cardiomyopathy (TICM) is typically reversible with rhythm control, but individual susceptibility remains poorly understood and may reflect genetic predisposition.

**Case Summary:**

A 66-year-old woman with paroxysmal atrial fibrillation (AF) presented with new-onset heart failure. Genetic testing identified a likely pathogenic heterozygous *ABCC9* gene variant (c.3892+2T>C), not previously associated with dilated cardiomyopathy or AF. *ABCC9* loss-of-function mutations have been linked with cardiac channelopathies and cardiomyopathies. Ventricular function improved with rhythm control and medical therapy.

**Discussion:**

This case illustrates the potential role of *ABCC9* mutations in arrhythmia-induced cardiomyopathy beyond pure TICM. Although this variant has not been previously reported in affected individuals, existing models support its pathogenicity. The co-occurrence of prolonged QT, familial AF, and dilated cardiomyopathy underscores the value of genetic testing in cardiac disease.

**Take-Home Messages:**

Genetic testing may reveal causes in atypical or treatment-resistant cardiomyopathies and arrhythmias. This novel *ABCC9* variant suggests a genetic contribution to AF-induced cardiomyopathy beyond the expected course of TICM.

## Graphical Abstract

**Figure:**
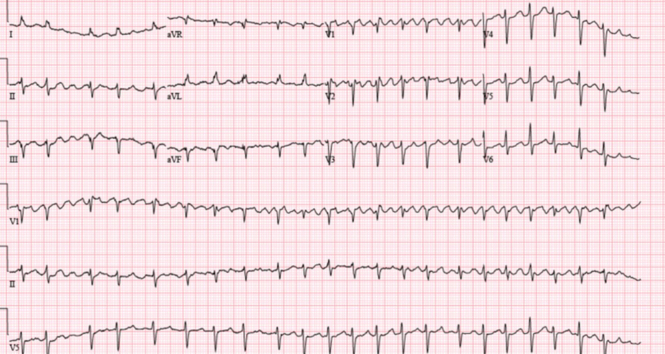

## History of Presentation

A 66-year-old diabetic woman with a 2-year history of paroxysmal atrial fibrillation (AF) presented with AF and rapid ventricular response (RVR). She had 2 weeks of progressively worsening dyspnea to minimal exertion, palpitations, orthopnea, and lower extremity edema, without chest pain or syncope. She had been strictly compliant with metoprolol and dabigatran. Her mother had AF at age 50, and her father had a pacemaker implanted at age 54. She smoked <100 lifetime cigarettes and reported no alcohol or recreational drug use. Her blood pressure was 106/72 mm Hg, heart rate was 102 beats/min, and respiratory rate was 18 breaths/min. Heart sounds were irregularly irregular, without murmur. She had bilateral lung crackles, jugular venous distension to the jawline, and bilateral lower extremity edema. There was no palpable hepatosplenomegaly or signs of chronic liver disease.

**Take-Home Messages**

• Genetic testing may uncover underlying causes in atypical or treatment-resistant cardiomyopathies and arrhythmias.
• The identification of a novel *ABCC9* variant highlights the potential role of genetic mutations in AF-induced cardiomyopathy, particularly when features exceed those typical of TICM.

## Past Medical History

No stroke, myocardial infarction, chemotherapy, radiation, or toxic exposures occurred in the past.

## Differential Diagnosis

Initial considerations included acute heart failure due to ischemic heart disease, arrhythmia-induced cardiomyopathy, familial dilated cardiomyopathy, and congenital heart disease.

## Investigations

**Figure 1:**
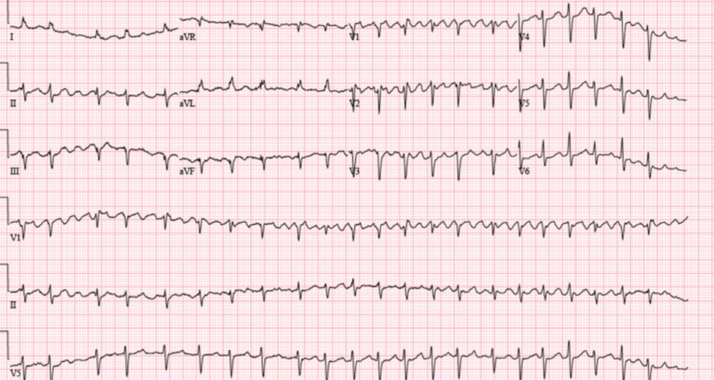
Electrocardiogram Showing Atrial Fibrillation With Rapid Ventricular Response and Prolonged QTc (531 ms)

**Figure 2:**
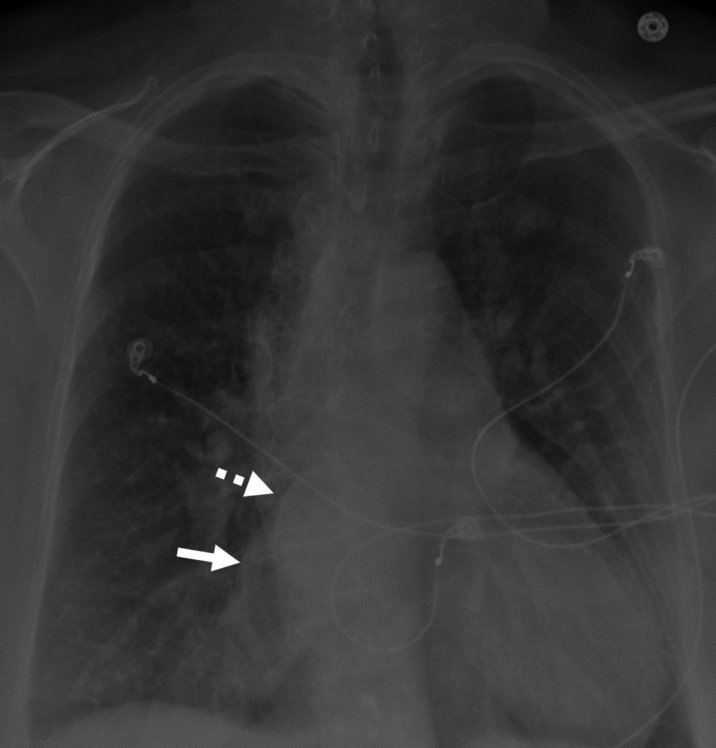
Anteroposterior Chest Radiograph Showing Bilateral Interstitial Pulmonary Congestion and Left (Dashed Arrow) and Right (Solid Arrow) Atrial Enlargement

**Figure 3:**
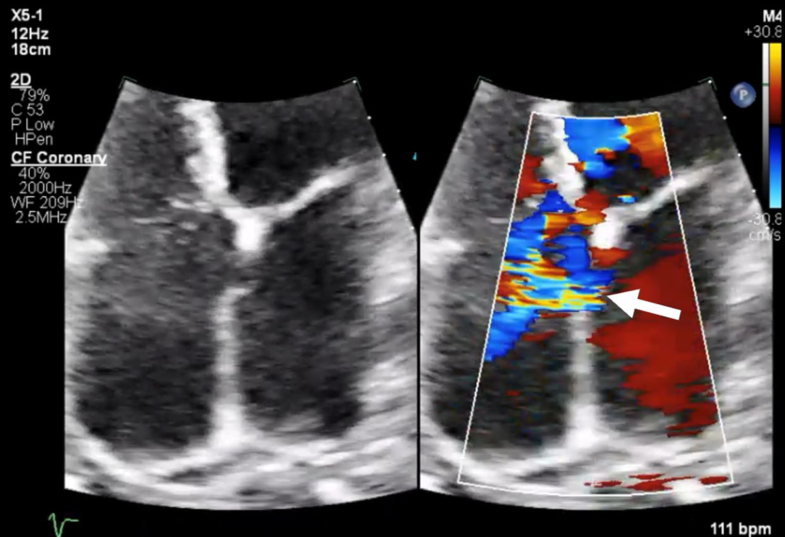
Transthoracic Echocardiogram 4-Chamber View Showing Left-to-Right Atrial Shunt by Color Doppler (Arrow)

Electrocardiogram (Figure 1) demonstrated AF with RVR and prolonged QTc of 531 ms. A recent 48-hour Holter monitor showed an AF burden of 46% (maximum episode duration: 22 hours). Chest x-ray revealed mild bilateral interstitial pulmonary edema and biatrial enlargement (Figure 2). Transthoracic echocardiography showed a left ventricular ejection fraction (LVEF) of 20% to 24%, right ventricle dilation, and a secundum atrial septal defect (ASD) (Figure 3). Pharmacological nuclear stress test within the past 2 years revealed no evidence of stress-induced ischemia and estimated a normal LVEF of 60%.

Given her low LVEF, ongoing AF, and normal prior nuclear stress test, tachycardia-induced cardiomyopathy (TICM) was suspected. She was treated with intravenous loop diuretics and was scheduled for a transesophageal echocardiogram and direct-current cardioversion with plans to initiate antiarrhythmic therapy to maintain sinus rhythm. Transesophageal echocardiogram confirmed a large secundum ASD with left-to-right shunting.

## Management

The patient was successfully cardioverted with a single synchronized 300-J biphasic shock. Postcardioversion electrocardiogram showed sinus rhythm with a QTc of 502 ms. She was discharged home the next day on low-dose guideline-directed medical therapy, including metoprolol, losartan, empagliflozin, and spironolactone.

## Outcome and Follow-Up

Within 1 week, AF with RVR and heart failure symptoms recurred. Repeat 2-week Holter monitoring demonstrated persistent AF, with average heart rate of 110 beats/min. Given her persistently low LVEF, she underwent left and right heart cardiac catheterization to rule out multivessel coronary artery disease. Angiography revealed normal coronary arteries, low systemic vascular resistance of 542 dyn⋅s/cm^5^, and an oxygenation step-up at the low right atrium, with a pulmonary-to-systemic flow ratio of 2.14, consistent with a significant interatrial left-to-right shunt (Table 1).

**Table 1:** Left and Right Catheterization Findings 1 Week After Discharge

	Patient Values	Abnormal Values
Right atrial pressure	8 mm Hg	>10-30 mm Hg
Right ventricular systolic pressure	27 mm Hg	>40 mm Hg
Right ventricular end-diastolic pressure	4 mm Hg	>5 mm Hg
Pulmonary artery systolic pressure	24 mm Hg	>30 mm Hg
Pulmonary artery diastolic pressure	10 mm Hg	>12 mm Hg
Pulmonary artery mean pressure	18 mm Hg	>18 mm Hg
Pulmonary capillary wedge pressure	10 mm Hg	>12 mm Hg
Systemic vascular resistance	542 dyn⋅s/cm^5^	<700 to >1,500 dyn⋅s/cm^5^
Pulmonary-to-systemic flow ratio (Qp/Qs)	2.14	<1:1 and >1:1

Green text indicates normal value; Yellow text indicates low value; Orange text indicates high value.

**Figure 4:**
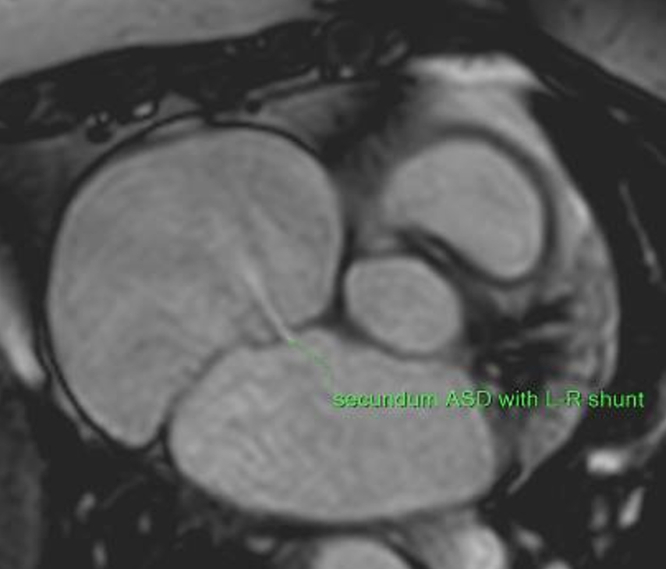
Cardiac Magnetic Resonance Cine Imaging Showing a 13 × 6 mm Secundum Atrial Septal Defect With Left-to-Right Shunt

Cardiac magnetic resonance imaging confirmed a large secundum ASD (13 × 6 mm) with left-to-right shunt, dilated right ventricle, and LVEF of 29%. There was no late gadolinium enhancement to suggest infiltrative cardiomyopathy, prior myocarditis, or infarct (Figure 4).

Further titration according to guideline-directed medical therapy was not tolerated. Rhythm control proved difficult because of the prolonged QTc, making the safe use of antiarrhythmics unfeasible. A surgical approach was preferred given multiple planned interventions and her overall low surgical risk. A biatrial Cox-maze IV lesion set was performed using bipolar radiofrequency ablation clamp and cryothermal ablation. The left atrial appendage was clipped, and the ASD was directly sutured without residual shunt.

Despite successful sinus rhythm restoration, QTc remained prolonged, prompting evaluation for long QT syndrome. Genetic testing revealed a heterozygous splice-site variant in *ABCC9* (c.3892+2T>C), which was classified as likely pathogenic. Whole mitochondrial genome sequencing and testing of her asymptomatic children returned negative results. At follow-up, the patient developed frequent, prolonged sinus pauses consistent with sinus node dysfunction (SND) requiring dual-chamber pacemaker implantation. LVEF improved to 40% to 45%, with resolution of heart failure symptoms and a reduction in AF burden (<1%).

## Discussion

This patient presented with nonischemic cardiomyopathy, persistent AF, and a prolonged QT interval—initially attributed to TICM. Arrhythmias often coexist with heart failure and left ventricular dysfunction. Although TICM is typically reversible with restoration of sinus rhythm—confirmed by LVEF recovering within 6 months—the factors predisposing certain patients to TICM remain unknown. It is often difficult to determine whether the cardiomyopathy results from sustained tachycardia or if the tachycardia is a consequence of low cardiac output due to the cardiomyopathy itself.

Cardiac magnetic resonance imaging helped to rule out infiltrative cardiomyopathy and obtain accurate right ventricle dimensions to further support the indication for ASD closure. Late gadolinium enhancement and T1-mapping are essential for detecting myocardial fibrosis.^1^ In the absence of late gadolinium enhancement, T1-mapping can assess for diffuse fibrosis. In this case, T1 estimation was inaccurate because of artifact from image acquisition during AF,^2^^,^^3^ so a degree of diffuse fibrosis could not be excluded.

Although our patient showed improvement in LVEF, full recovery was not achieved, possibly because of residual ongoing AF burden, cardiac structural remodeling, ventricular fibrosis, or underlying genetic predisposition.^4^^,^^5^ This suggests a multifactorial etiology, and arrhythmia-induced cardiomyopathy (AICM) may better explain her presentation. AICM is regarded as a distinct entity from TICM and is characterized by ongoing left ventricular dysfunction in patients with AF, despite adequate rate control. Proposed mechanisms for AICM include impaired calcium dynamics, loss of atrial contractility leading to increased sympathetic activity, and aggravated diastolic dysfunction.^6^ Further investigation of atrial fibrosis and inflammation could shed light on the pathophysiology of AF-induced cardiomyopathy.

AF has been described as a complication of percutaneous ASD closure in numerous trials; however, neither surgical nor percutaneous closure of ASD eliminates AF. One study reported concomitant AF in 29% of patients with noniatrogenic ASD and noted that the presence of ASD in patients with AF was associated with a higher rate of hospitalization, heart failure, pacemaker implantation, and difficulties in maintaining sinus rhythm.^7^ In our patient, the ASD may partially explain her clinical presentation, especially when considering the potential for AF to initiate or exacerbate AICM. Additionally, the persistence of a prolonged QT and the identification of an *ABCC9* variant raises questions about a possible genetic cause.

Several mutations in cardiac transcription factor genes, such as *NKX2-5*, *GATA4*, and *TBX5*, have been linked with ASD.^8^ The *ABCC9* gene, which encodes the sulfonylurea receptor 2 subunit of the cardiac ATP-sensitive potassium channel, has been associated with several cardiac channelopathies, including autosomal dominant Cantú syndrome, familial AF, and dilated cardiomyopathy.^9^ However, isolated ASD is not a defining feature of Cantú syndrome based on the current literature.^10^ This suggests that other mechanisms, such as underlying ion channel dysfunction or metabolic stress response, could be at play in our patient's presentation.

The *ABCC9* gene plays a critical role in myocardial repolarization and metabolic stress response. Loss-of-function variants have been implicated in dilated cardiomyopathy and familial AF,^11^, ^12^, ^13^ whereas gain-of-function mutations cause Cantú syndrome.^10^ The identified splice-site variant (c.3892+2T>C) is predicted to disrupt normal RNA splicing, likely leading to a nonfunctional protein.^14^ Although this specific variant has not been previously reported in affected individuals, its classification as likely pathogenic reflects accumulating evidence that *ABCC9* loss-of-function mutations can contribute to cardiac dysfunction. Unlike genes such as *PITX2*, *TBX5*, or *SCN5a*, *ABCC9* has not been consistently implicated in genome-wide association studies (GWAS),^15^ likely owing to limited power to detect rare variants.

A surgical approach for rhythm control was carefully selected after discussion with the heart team and considering the patient's preferences, low surgical risk, and indications for ASD closure, pulmonary vein isolation, and left atrial appendage exclusion. Although trans-septal puncture in the presence of atrial septal occluder devices has been safely performed,^16^ concerns with this singular approach favored surgical ASD closure, allowing an easier avenue for future catheter ablation if needed.

Our patient had multiple risk factors for SND, which is commonly encountered in patients with AF and is often unmasked after AF ablation or cardioversion. Progressive atrial remodeling and fibrosis from persistent AF is known to contribute to the pathogenesis of SND.^17^ Surgical AF ablation itself, with or without concomitant valve surgery, has also been associated with an increased (albeit small) risk of requiring a pacemaker.^18^ Moreover, a genetic predisposition to SND is plausible. Variants expected to result in loss of function in the *ABCC9* gene encoding for SUR2 potassium channels have been linked to diminished sinoatrial node automaticity in murine models,^19^ and advanced atrioventricular block in the absence of structural heart disease was described in a young male patient.^20^

## Conclusions

Our case raises the possibility that this genetic variant contributed to our patient's cardiomyopathy, particularly in the context of AICM not fully explained by tachyarrhythmia alone. While the findings suggest a potential link to *ABCC9*-related disease, further studies—including family screening and analysis of large-scale genomic databases (eg, gnomAD, ClinVar)—are needed to clarify the pathogenicity of this and similar variants. This case illustrates the value of genetic testing in identifying the etiology of unexplained cardiomyopathy and arrhythmias. Early recognition of *ABCC9* mutations could have significant implications for diagnosis and treatment, guiding clinicians by revealing genetic drivers of arrhythmia and myocardial dysfunction.

## Funding Support and Author Disclosures

This material is the result of work supported with resources and the use of facilities at the Orlando VA Healthcare System (Orlando, Florida, USA). The contents of this publication do not represent the views of the Department of Veterans Affairs or the United States Government. The authors have reported that they have no relationships relevant to the contents of this paper to disclose.
